# HPLC Analysis of Phenols in Negroamaro and Primitivo Red Wines from Salento

**DOI:** 10.3390/foods8020045

**Published:** 2019-02-01

**Authors:** Andrea Ragusa, Carla Centonze, Maria E. Grasso, Maria F. Latronico, Pier F. Mastrangelo, Federica Sparascio, Michele Maffia

**Affiliations:** 1 Department of Engineering for Innovation, University of Salento, via Monteroni, 73100 Lecce, Italy; 2 CNR Nanotec, Institute of Nanotechnology, via Monteroni, 73100 Lecce, Italy; 3 Department of Biological and Environmental Sciences and Technologies, University of Salento, via Monteroni, 73100 Lecce, Italy; carla_centonze@libero.it (C.C.); nutrizionegrasso@gmail.com (M.E.G.); latronico-francesca@libero.it (M.F.L.); mastrangelo.pf@hotmail.it (P.F.M.); federicasparascio@alice.it (F.S.)

**Keywords:** antioxidants, wine, phenols, hydroxytyrosol, quercetin, *trans*-resveratrol, HPLC

## Abstract

Wine is probably the oldest and still most consumed alcoholic beverage in the world. Nevertheless, it contains several biomolecules with beneficial health effects. Phenols are among them and, in this article, we identified and quantified by HPLC catechin, gallic acid, hydroxytyrosol, quercetin, *trans*-resveratrol, and syringic acid in Primitivo and Negroamaro red wines from Salento, in Southeast Italy. The concentrations of the analyzed antioxidant molecules were quite high in all varieties. Gallic acid and catechin were the most abundant, but significant concentrations of quercetin, hydroxytyrosol, syringic acid, and *trans*-resveratrol were also found. Multivariate statistical analysis was also employed to discriminate between Negroamaro and Primitivo wines, suggesting the variables influencing their separation.

## 1. Introduction

The beneficial properties of the Mediterranean diet are well recognized, and they have also been related, among many other bioactive compounds from other foods, to the antioxidant properties of two of its fundamental constituents, i.e., wine and extra-virgin olive oil [1,2,3,4]. The antioxidant efficacy of these products is due to the presence of strictly related, although different, classes of organic molecules mainly derived from phenols. These types of molecules have been shown to positively interfere in a multitude of biological processes by protecting the organism through oxidative stress of, for example, low-density lipoproteins (LDLs) and DNA. This results in potential beneficial effects on the vascular system, on atherosclerosis, and on the development of some age-related cancers, just to cite a few [5,6,7]. However, polyphenols are not produced in animals, and humans have to introduce these compounds and aromatic amino acids through the diet.

Although phenolic compounds are the third-most abundant species in grapes, their type and concentrations vary considerably because of genetic factors, i.e., grape type, and environmental factors. Furthermore, production techniques and conditions used can also lead to significant differences in the final product [8,9].

Flavonoids, such as anthocyanins and tannins, and nonflavonoids, such as hydroxystilbenes and phenolic acids, are the two major categories of polyphenols, although many subclasses are present depending on their chemical structure. Gallic acid is an hydroxybenzoic acid that has been reported to inhibit the formation of amyloid fibrils by α-synuclein, thus having a potential neuroprotective effect [10]. Syringic acid has a similar structure, although methylated on two hydroxylic groups, and it recently was shown to be able to reduce the inflammatory response of asthma in mice models [11]. Quercetin belongs to flavonols, and it has received considerable attention because it is believed to prevent lipid peroxidation, thus protecting against several degenerative diseases [12]. Catechin is a flavanol derivative that has been reported to have a vasodilatation effect, thus contributing to blood pressure regulation [13,14]. Because of its strong antioxidant effect, the simple molecule of hydroxytyrosol has been widely studied in the literature for treating osteopathy, cancer, and cardiovascular and neurodegenerative diseases [15,16,17,18]. Similarly, the stilbene *trans*-resveratrol has been extensively studied because of its cardioprotective, chemopreventive, anti-inflammatory, neuroprotective, and anti-angiogenic activities, just to name a few [19,20,21,22,23].

Apulian wines are among the most diffused and exported Italian wines. Nevertheless, despite their importance, not much data is available in the literature. Most studies have involved the characterization of red wines through chemical methods or by chromatographic and spectroscopic techniques, and the results have also been exploited to identify grape varieties and optimize production procedures [24,25,26,27,28,29,30]. We have already studied the phenolic composition of several red, rose, and white wines from Apulia, and the red ones had the highest concentration of antioxidant molecules, and thus potentially the highest beneficial health effects [4]. We now focus our attention on investigating the content of polyphenols only in these types of wines, and in particular in those obtained from Negroamaro and Primitivo di Manduria cultivar. Negroamaro is an autochthonous grape variety grown exclusively in Salento, and literally means “black” and “bitter” because of its organoleptic characteristics. Primitivo di Manduria is another autochthonous grape quite similar to the Californian Zinfandel, with which it shares the same origin, and it is called “primitive” because of the characteristic early maturation of its vine compared to other Apulian varieties. Wines produced from these varieties are probably the most widespread Apulian wines, and they are also very important from an economic point of view, due to them being exported worldwide. This is why we believe that a deeper understanding of their chemical characteristics is of paramount importance from a nutraceutical point of view.

## 2. Materials and Methods

### 2.1. Chemicals

Standards of catechin, gallic acid, hydroxytyrosol, quercetin, and syringic acid were purchased from Sigma-Aldrich (Milan, Italy), and *trans*-resveratrol was purchased from Dr. Ehrenstorfer GmbH (Augsburg, Germany). Ethanol and methanol (both HPLC-grade) and acetic acid were purchased from J.T. Baker (Deventer, the Netherlands). Water (HPLC-grade) was purchased from Carlo Erba Reagenti (Milan, Italy).

### 2.2. Samples

**Figure 1:**
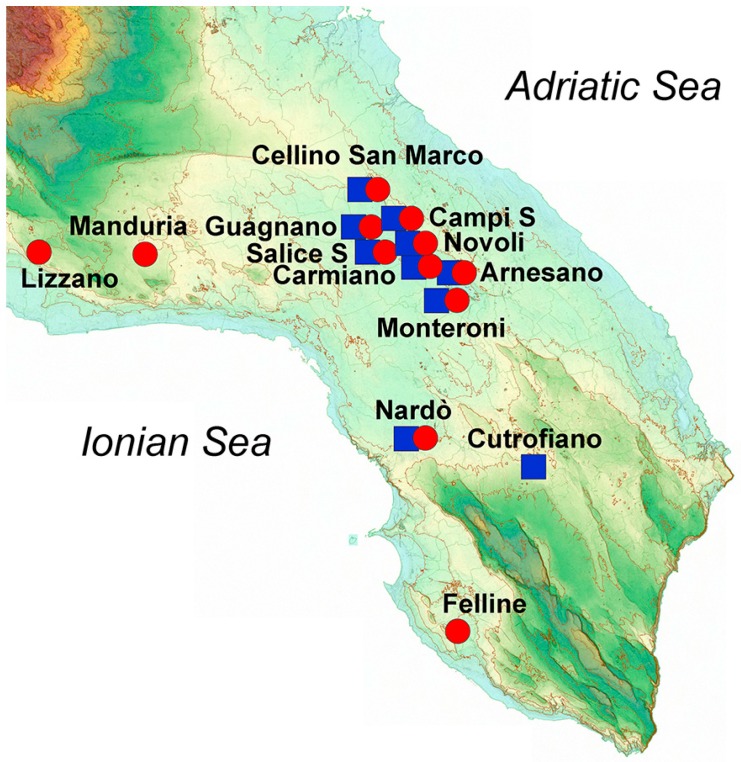
Image representing the orographic features of Salento and the sites of production of the wines studied. Red circles represent Negroamaro wines, and blue squares represent Primitivo wines.

The most characteristic red *Vitis vinifera* grapes from Salento (see Figure 1 for details about the specific location) were employed for the production of the wines analyzed, i.e., Negroamaro (17 samples) and Primitivo (14 samples), for a total of 31 samples. All wines were produced in 2015, and the samples were acquired directly from the final commercially available bottles, all having a Controlled Designation of Origin (DOC) certification, which guarantees their designation of origin. Table 1 reports the details of the number of samples, the average alcoholic grade (based on the data reported on the label of the bottles), and the average phenolic concentration, in mg/kg of wine, grouped for type of wine.

### 2.3. HPLC Analysis

An aliquot of wine (5 mL) was collected directly from each bottle, filtered by using a 0.45-µm pore size regenerated cellulose filter (VWR International, Milano, Italy), and analyzed without further purification. The analytical system was composed of a High-Performance Liquid Chromatography 1220 Infinity system with an autosampler (Agilent Technologies, Palo Alto, CA, USA) interfaced with a diode array detector (model G1315B DAD system, Agilent, Palo Alto, CA, USA). An Eclipse Plus C18 column (particle size 5 µm, 4.6 × 250 mm, Agilent, Palo Alto, CA, USA) was used as a stationary phase, and a mixture of water/methanol/acetic acid in different ratios as a mobile phase (solvent A: 75:20:5, v/v/v; solvent B: 50:45:5, v/v/v). The gradient conditions were as follow: 0% B at 0 min, 100% B at 30 min, 100% B at 40 min, 0% B at 50 min. The system conditions were as follows: Eluent flow at 1.0 mL/min, column temperature at 25 °C, sample injection of 20 µL. Catechin, gallic acid, hydroxytyrosol, and syringic acid were detected at 280 nm, while *trans*-resveratrol and quercetin were detected at 309 nm. Obtained retention times were gallic acid at 3.3 min, hydroxytyrosol at 4.3 min, catechin at 5.4 min, syringic acid at 10.1 min, *trans*-resveratrol at 24.8 min, and quercetin at 34.8 min. The limit of detection (LOD) and quantification (LOQ) were above 0.08 and 0.24 µg/mL, respectively, for all analytes. Standard solutions at increasing concentrations of the analytes in ethanol were prepared and used for obtaining the corresponding calibration curves, which were fitted to a linear equation with zero intercept (R^2^ > 0.98 for all samples). The concentrations of the analyzed phenols in the wine samples were determined by interpolating the corresponding peak areas, and each sample was run in triplicate. The raw chromatographic data are available upon request from the Multilab-Chamber of Commerce of Lecce (multilab@le.camcom.it).

### 2.4. Statistical Analysis

**Figure 2:**
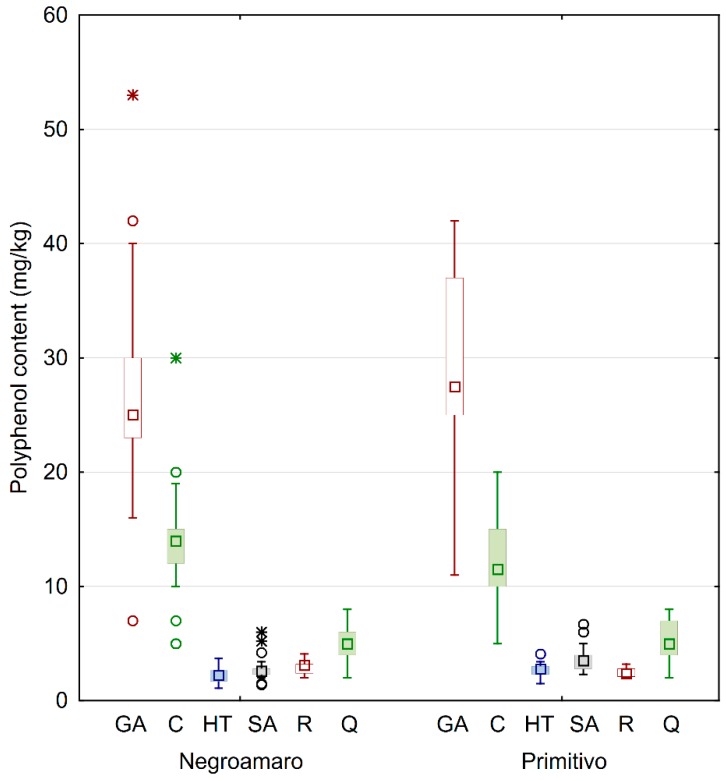
Box and whiskers plot of the analyzed phenols grouped by type of wine. The squares inside of boxes are median values. The height of the box is equal to the interquartile distance, indicating the distribution for 50% of the data. The whiskers outside the box represent non-outlier data, while circles and stars represent outlier and extreme values, respectively. GA: gallic acid; C: catechin; HT: hydroxytyrosol; SA: syringic acid; R: *trans*-resveratrol; Q: quercetin.

The reported concentrations represent the mean values for each specific type of wine. The standard deviations represent the difference among different samples from the same category. The standard deviation for replicates of the same sample was always <5%. Obtained polyphenolic values were rounded to one decimal place. Lower box values (LBVs) and upper box values (UBVs) in Figure 2 were defined as the 25th and 75th percentiles, respectively. Outliers and extremes were defined according to the formula:

data point value > of *UBV* + *C* × (*UBV* − *LBV*) (1)

data point value < of *LBV* − *C* × (*UBV* − *LBV*) (2)

where *C* is a coefficient defined as 1 for whiskers, 1.5 for outliers, and 3 for extremes.

Univariate statistical analyses (fold-change (FC), Log2(FC), *t*-test, false discovery rate (FDR), and Pearson correlation analysis) were performed using MetaboAnalyst (www.metaboanalyst.ca). Multivariate statistical analysis (MVA) was performed using SIMCA 14.1 software (MKS Umetrics, Malmö, Sweden). Principal component analysis (PCA) and orthogonal partial least-squares discriminant analysis (OPLS-DA) were performed using the detected polyphenols as variables (*n* = 6) and the wine samples as observations (*n* = 31), yielding a matrix of 186 data points, while grape type was used as qualitative information (classes). Strong outliers were removed from the computation to obtain a better fit. *R*^2^_X_(cum) and *R*^2^_Y_(cum) were used as parameters for describing the goodness of the fit, while *Q*^2^(cum) was used to describe the predictive ability of the model.

## 3. Results and Discussion

The content of several polyphenols, namely gallic and syringic acid, catechin, hydroxytyrosol, quercetin, and *trans*-resveratrol, was quantified in different types of commercially available wines from Salento, a geographical region in Southern Apulia, in Italy, and the results are shown in Table 1.

The data represent the average values and the corresponding standard deviations of the detected molecules for all the samples and grouped for type of wine. Negroamaro and Primitivo wines were prepared from the corresponding grapes grown locally and from pure autochthonous varieties. Production year was 2015 for all samples, and the bottles from which the samples were drawn had a DOC designation, which is a quality assurance label for Italian wines. Reported average alcoholic grade was almost 14% for both wines, with slightly higher values for Primitivo wines (average 14.0% ± 0.6%) and lower ones for Negroamaro samples (13.5% ± 0.6%). These values were quite similar to those reported in previous years (2007–2013) for the same type of wines from Salento, indicating poor variability in the sugar content of the grapes despite potential yearly variations in the environmental conditions, assuming similar production processes [4]. Nevertheless, these wines are well known for having quite high alcoholic content, which is also one of the reasons they are widely implemented in blend wines, i.e., to increase the final alcoholic content.

All of the 31 commercially available samples (17 Negroamaro and 14 Primitivo bottles) showed significant average amounts of the investigated polyphenols (Table 1). Gallic acid was the most abundant analyzed antioxidant molecule (about 29 ± 10 mg/kg), followed by catechin with almost half that concentration (about 13 ± 5 mg/kg). Much lower concentrations of quercetin were found (about 5 ± 2 mg/kg) and even less of hydroxytyrosol and syringic acid (about 3 ± 1 mg/kg for both analytes). The average concentration of *trans*-resveratrol was almost 3 mg/kg, quite a significant amount considering its beneficial effect. However, it is not surprising if we take into account that all samples were from red wines, much richer in this biomolecule compared to white and rosé wines, and that these values were in line with already reported values [4,30].

In general, calculated standard deviations for each category were quite high, probably because of the many variables involved before getting to the commercialized wines that were analyzed. In fact, the samples were collected from bottles of different wineries, and as such they came from different geographical areas (see Figure 1 for details). Similarly, other factors involved in the production process likely influenced the concentration of the secondary metabolites, thus yielding wines with different quality. This behavior can be better understood by looking at the box and whiskers plot in Figure 2, also showing median values, extremes, and outliers grouped by wine category.

The results clearly indicate that gallic acid was the most abundant phenol in all wines, and there was no large difference when comparing the grape type. In fact, Negroamaro and Primitivo wines averaged about 28 and 29 mg/kg, respectively. On the other hand, the homogeneity of the samples yielded quite high variability, especially in Primitivo wines, while the concentrations of gallic acid in Negroamaro were more similar, as observed by the interquartile distance, although few outliers were found. A similar behavior was also observed for catechin in Negroamaro and Primitivo wines (about 14 and 12 mg/kg, respectively), while quercetin values were much more similar. The quantities of the remaining analyzed polyphenols, i.e., hydroxytyrosol, syringic acid, and *trans*-resveratrol, were considerably smaller compared to the already discussed antioxidants. Syringic acid was slightly higher in Primitivo samples compared to Negroamaro (about 4 and 3 mg/kg, respectively). Finally, all red wines also contained good amounts of hydroxytyrosol and *trans*-resveratrol (almost 3 mg/kg each), with the former being more abundant in Primitivo wines and the latter in Negroamaro ones.

The concentrations of the phenols detected in these wines were much lower compared to those employed in the studies that evidenced their potential therapeutic effects. However, the regular consumption of moderate quantities of these wines might also lead to beneficial effects, as assumed in the French paradox.

Compared to our previous results on similar wines from past years, comparable amounts of phenols were mostly obtained on average, and only the concentration of syringic acid was significantly lower in both wines [4]. Individually, the most striking differences were noted in the Primitivo samples, which had significantly higher concentrations of gallic acid and only slightly more quercetin. On the other hand, Negroamaro wines had much less variability compared to previous vintages, but only a modest increase in gallic acid and slightly lower hydroxytyrosol.

Several environmental factors (nutrients in soil, water, temperature, sunlight exposure, etc.), as well as differences in the production process, might have positively or negatively altered the content of the phenols in wines. In this study, we analyzed wines exposed to external factors comparable to those of the wines previously reported. Nevertheless, a more specific investigation is needed in order to be able to draw some conclusion that could be exploited in viticultural practices, and this might be the object of future studies.

**Figure 3:**
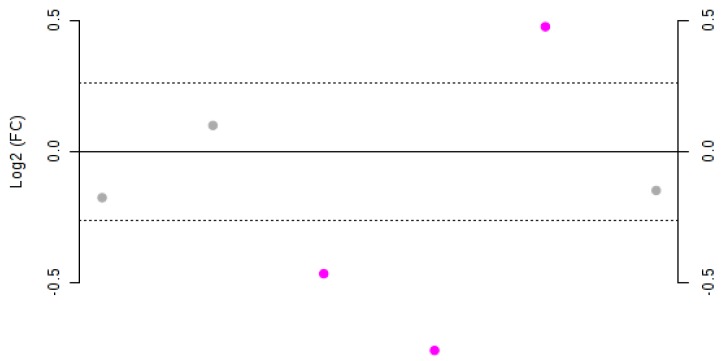
Fold-change (FC) plot of the Log2 values for Negroamaro and Primitivo wine samples. Dots represent, from left to right, gallic acid, catechin, hydroxytyrosol, syringic acid, *trans*-resveratrol, and quercetin. Values with a Log2(FC) > 1.2 are highlighted in purple.

In order to highlight significant differences between concentrations of the phenols in Negroamaro and Primitivo wines, a fold-change (FC) analysis was performed (Figure 3).

Gallic acid, catechin, and quercetin did not show significant variation (Log2(FC) < 1.2) between the two species. On the other hand, hydroxytyrosol, syringic acid, and *trans*-resveratrol were significantly different, as also confirmed by *t*-test analysis (Table 2). In particular, Negroamaro wines were shown to have statistically significant higher amounts of *trans*-resveratrol, while the concentration of hydroxytyrosol and syringic acid was statistically greater in Primitivo samples, as confirmed by both *p*- and FDR values.

**Figure 4:**
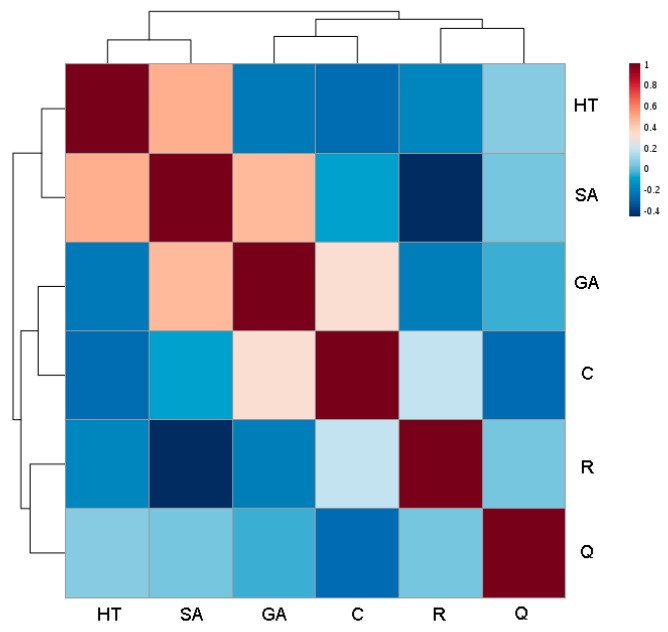
Correlation heatmap of the analyzed polyphenols in Negroamaro and Primitivo red wines from Salento. A red color indicates a positive correlation, while a blue one a negative correlation (the darker the color, the higher the correlation coefficient (as reported in the column to the right)). GA: gallic acid; C: catechin; HT: hydroxytyrosol; SA: syringic acid; R: *trans*-resveratrol; Q: quercetin.

The behavior of the phenols in Negroamaro and Primitivo wines was also investigated by correlation analysis to check if any pattern could be observed through some kind of metabolite-metabolite relationship (Figure 4).

A strong negative correlation was observed between *trans*-resveratrol and syringic acid (−0.446), which were in fact detected in high concentrations in Negroamaro and Primitivo wines, respectively. On the other hand, a strong positive correlation was noted between syringic acid and hydroxytyrosol and gallic acid (0.490 and 0.457, respectively), but not between hydroxytyrosol and gallic acid (−0.218). These correlations might have been due to implicit characteristics of the two varieties as well as to influences of external factors that can favor or disadvantage the growth of the secondary metabolites. Nevertheless, further studies are needed to validate and understand these behaviors, and how external factors can alter, and hopefully improve, the concentration of these antioxidants with important health beneficial effects.

### 3.1. Multivariate Statistical Analysis

The concentrations of the polyphenols quantified in the Negroamaro and Primitivo samples were exploited in subsequent statistical multivariate analysis to investigate any unsupervised and supervised clustering of the two types of wine.

**Figure 5:**
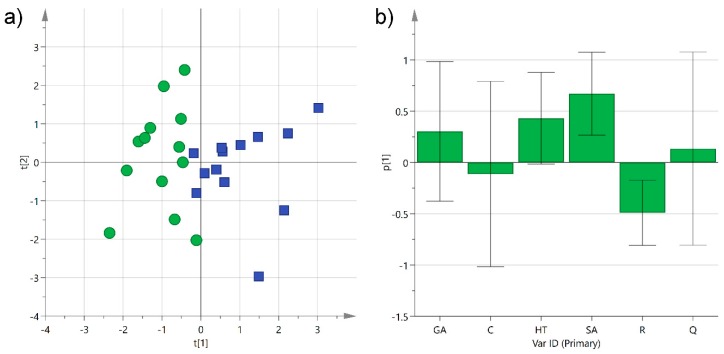
(**a**) Principal component analysis (PCA) score plot of PC1 (*R*^2^_X_ 0.29) versus PC2 (*R*^2^_X_ 0.25) for Negroamaro (green circles) and Primitivo (blue squares) wine samples; (**b**) histogram plot for the loadings of PC1. GA: gallic acid; C: catechin; HT: hydroxytyrosol; SA: syringic acid; R: *trans*-resveratrol; Q: quercetin.

Principal component analysis (PCA) was first performed separately on the two categories to check the homogeneity of the samples. After removing three outliers, probably because of the variability introduced by different environmental conditions and production techniques employed, the two classes showed a satisfactory grouping with an R^2^_X_ of 0.54 and 0.61 for Negroamaro and Primitivo, respectively. The same unsupervised analysis was then performed on both groups together, and the obtained first two components were able to describe 54% of the sample variability. The corresponding scatter score plot showed good separation of the two types of wine on the first component (explaining 29% of the data alone), while samples were more spread out on the second one (Figure 5a). Analysis of the histogram plot representing the loadings of the variables for PC1 evidenced that hydroxytyrosol and syringic acid in Primitivo wines, and *trans*-resveratrol in Negroamaro ones, contributed to the separation of the two groups (Figure 5b). On the other hand, gallic acid, catechin, and quercetin did not exceed significantly in any of the samples, and they did not help in clustering the wine samples.

**Figure 6:**
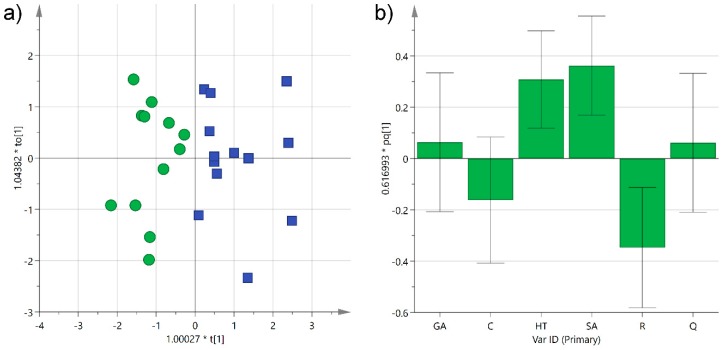
(**a**) Orthogonal partial least-squares discriminant analysis (OPLS-DA) score plot of PC1 (*R*^2^_X_ 0.33) versus PC2 (*R*^2^_X_ 0.26) for Negroamaro (green circles) and Primitivo (blue squares) wine samples; (**b**) histogram plot for the loadings of the first predictive component of the model. GA: gallic acid; C: catechin; HT: hydroxytyrosol; SA: syringic acid; R: *trans*-resveratrol; Q: quercetin.

A further investigation was performed using orthogonal partial least-squares discriminant analysis (OPLS-DA) in order to obtain a predictive model that could be used to determine the grape type in unknown samples. As expected, even better grouping was obtained with the supervised analysis, and the two categories separated completely along the first component, as observed in the scatter score plot (*R*^2^_X_(cum) 0.59; *R*^2^_Y_(cum) 0.71) (Figure 6a). As already obtained by the unsupervised model, *trans*-resveratrol, and to a minor extent catechin, were shown to contribute the most to the separation of Negroamaro samples, while syringic acid and hydroxytyrosol to that of Primitivo wines, as indicated by the coefficients of the histogram plot corresponding to the first predictive component (Figure 6b). On the other hand, gallic acid and quercetin were equally distributed in the two categories. The OPLS-DA also yielded a satisfactory predictive model, with a cumulative *Q*^2^ of 0.57 obtained by cross-validating the data, which could be used to classify unknown samples. Furthermore, this model could be easily improved in its precision and accuracy by increasing the number of samples or the number of phenols analyzed, thus leading to potential practical use in the traceability of these wines and in discovering their adulteration.

Geographical features such as orography, type of terrain, climate, weather, and rainfall, as well as other artificial factors such as fertilization and irrigation, can largely influence the final characteristics of the harvested grapes and thus the quality of the obtained wine. In this regard, Salento is a relatively flat peninsula between the Adriatic Sea, facing the Strait of Otranto, and the Ionian Sea, facing the Gulf of Taranto (Figure 1).

**Figure 7:**
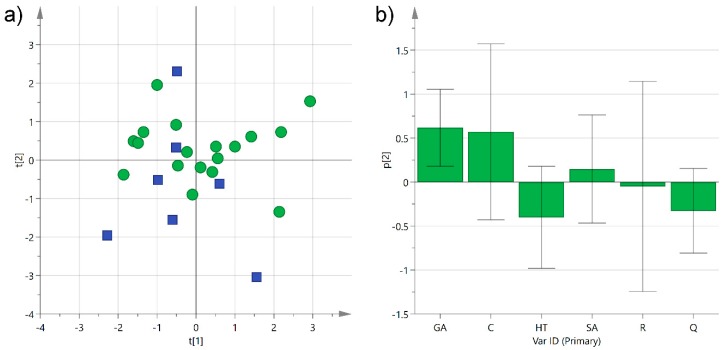
(**a**) PCA score plot of PC1 (*R*^2^_X_ 0.30) versus PC2 (*R*^2^_X_ 0.23) for Negroamaro and Primitivo wine samples categorized by their geographical location, either in the central mainland (green circles) or close to the Ionian Sea (blue squares); (**b**) histogram plot for the loadings of the second component of the model. GA: gallic acid; C: catechin; HT: hydroxytyrosol; SA: syringic acid; R: *trans*-resveratrol; Q: quercetin.

We have already observed how plants from Salento growing either in the central mainland or close to the sea produced different concentrations of phenols in extra-virgin olive oils [3]. In this case, the grape cultivars were grown principally in the central mainland of Salento (23 samples), especially close to the Lecce area, while only about 20% of the analyzed wines came from grapes cultivated near the Ionian Sea (eight samples), and none close to the Adriatic Sea. Among the latter, six out of eight wine samples were produced from Negroamaro grapes, while just two wine samples came from Primitivo grapes. Despite the variability among all the samples, a modest clustering could be observed when performing an unsupervised multivariate statistical analysis (Figure 7).

The PCA performed collectively on Negroamaro and Primitivo samples (*R*^2^_X_(cum) 0.53) showed some aggregation along the second component, with the samples produced in the central mainland predominantly arranged at positive or slightly negative values of the *y* axis, while samples coming from terrains close to the Ionian Sea clustered at slightly more negative values (excluding one sample). This partial separation was mainly caused by a higher concentration of gallic acid in the samples from the inner land, while the wines from wineries close to the Ionian Sea were somewhat richer in hydroxytyrosol and quercetin (Figure 7b). In order to improve separation and reduce the variability introduced by the different wine samples, the same type of analysis was performed on each category individually, i.e., Negroamaro or Primitivo, yielding similar trends, although the two models obtained were quite poor because of the low number of samples (data not shown). Despite the different type of matrix, we have already observed that extra-virgin olive oils produced from trees growing close to the Ionian Sea were richer in hydroxytyrosol than those coming from trees cultivated either in the central mainland or close to the Adriatic Sea, suggesting a potential influence of the terrain on the composition of the secondary metabolites [3]. Nevertheless, this type of investigation needs to be performed on a much larger and more homogeneous array of samples before any conclusion can be drawn.

## 4. Conclusions

Wine is an aliment with important nutraceutical properties mainly because of the presence of a variety of antioxidant molecules, also known as secondary metabolites. Some of these biomolecules were quantified in commercially available red wines from Salento, in Southeast Italy, through a reverse-phase chromatographic technique coupled with a UV-Vis detector. Negroamaro and Primitivo wines were shown to have quite high concentrations of the quantified phenols, especially gallic acid and catechin. Nevertheless, they also contained considerable amounts of quercetin, hydroxytyrosol, syringic acid, and *trans*-resveratrol. The results of this study help to characterize some of the nutraceutical properties of, probably, the most consumed and worldwide-exported local Apulian wines. Nevertheless, the results here presented can also be exploited to integrate already reported data in the literature in order to create a metabolic profile of these wines. The combined data could be exploited to generate a characteristic “fingerprint” of the wines through which a reliable multivariate statistical model could be obtained and exploited to guarantee the traceability and safety of these products [24,25].

## Figures and Tables

**Table 1:** Characteristics and content of phenols in wines (in mg/kg) organized by type of cultivar. ^1^

Grape Type	*n* of Samples	Alcoholic Grade (%)	Gallic Acid	Catechin	Hydroxytyrosol	Syringic Acid	*trans*- Resveratrol	Quercetin
Negroamaro	17	13.5 ± 0.6	28.2 ± 11.0	14.2 ± 5.6	2.3 ± 0.8	2.9 ± 1.2	3.0 ± 0.6	5.1 ± 1.6
Primitivo	14	14.0 ± 0.6	29.4 ± 8.6	12.6 ± 4.3	2.7 ± 0.7	3.8 ± 1.3	2.4 ± 0.4	5.4 ± 2.2
*Total*	31	13.8 ± 0.6	28.8 ± 9.8	13.4 ± 5.0	2.5 ± 0.8	3.4 ± 1.3	2.7 ± 0.5	5.3 ± 1.9

^1^ Values are expressed as mean ± standard deviation relative to the different wine bottles analyzed.

**Table 2:** Important phenols identified by fold-change analysis and *t*-test in Negroamaro and Primitivo wines. FDR: false discovery rate.

Phenols	FC	Log2(FC)	*p*-value	FDR
Syringic acid	0.591	−0.758	>0.001	0.001
*trans*-Resveratrol	1.391	0.476	>0.001	>0.001
Hydroxytyrosol	0.724	−0.466	0.005	0.010

FDR: false discovery rate; FC: fold-change.
